# Root characteristics of spring wheat under drip irrigation and their relationship with aboveground biomass and yield

**DOI:** 10.1038/s41598-021-84208-7

**Published:** 2021-03-01

**Authors:** Rui Chen, Xing-peng Xiong, Wen-han Cheng

**Affiliations:** Jingchu University of Technology, Jingmen, 448000 Hubei China

**Keywords:** Plant sciences, Plant physiology, Plant stress responses

## Abstract

The objectives of this two-year field experiment were (1) to study the effect of irrigation frequency and irrigation amount on the root characteristics of drip-irrigated spring wheat (*Triticum aestivum* L.) and (2) to determine the relationship between these root characteristics and aboveground biomass and yield. A split-plot design was used with two wheat cultivars (Xinchun 6 and Xinchun 22). The irrigation treatments consisted of three irrigation intervals (D_1,_ 13 d; D_2_, 10 d; and D_3_, 7 d) and three water amounts (W1, 3750 m^3^/ha; W2, 6000 m^3^/ha; and W3, 8250 m^3^/ha). The results showed that root length density (RLD) and root weight density (RWD) were greater at 0–20 cm than at 20–40 cm at flowering. The RLD was greater in D1 and D2 than in D3 in the shallow soil layer and did not differ among the treatments with different irrigation frequencies in deep soil. The RLD at the 0–20 cm depth of W3 was 17.9% greater than that of W2 and 53.8% greater than that of W1, and the RLD trend was opposite at the 20–40 cm depth. The root–shoot ratio was significantly higher in D2 than in the other treatment, whereas the RLD, RWD, leaf Pn and LAI were significantly greater in D3. Leaf Pn and LAI both increased as the irrigation amount increased. Regression analysis showed a natural logarithmic relationship between RWD and aboveground biomass (R^2^ > 0.60, *P* < 0.05) and binomial relationships of the RWD at 0–20 cm depth (R^2^ = 0.43, *P* < 0.05) and the RLD at 20–40 cm depth (R^2^ = 0.34, *P* < 0.05) with grain yield. We found that with the optimum irrigation amount (W2), increasing drip irrigation frequency can increase wheat root length and root weight and aboveground biomass accumulation, thereby improving yield and water use efficiency.

## Introduction

The availability of water for agriculture is decreasing worldwide. As a consequence, agricultural researchers are being asked to identify ways to produce more food with less water^1–3^. Drip irrigation is one of the best methods for improving agricultural water use efficiency (WUE)^4,5^. More than 3.7 million ha of cropland is drip irrigated worldwide^6^. Although most commonly used for horticultural crops, drip irrigation technology has now been adapted for many row crops, including cotton, maize, and wheat^7–10^.

Roots take up water and nutrients that are essential for crop growth. The importance of roots increases under water-limited conditions^11,12^. Root growth and spatial distribution are sensitive to soil water content^13^. Some drought-resistant crops overcome moisture stress by adjusting their root distribution when water availability is limited^14^. Irrigation methods have a significant effect on the root distribution in a soil profile^15^. In flood-irrigated systems, water is uniformly applied over a broad area of soil. This results in a deep and wide root distribution. In contrast, in drip-irrigated systems, water is applied to only a small part of the soil volume, and plant roots are mainly concentrated in this wetted part. Drip irrigation also has an effect on root distribution. In sweet corn, the root length density was found to be highest with surface drip irrigation at 0–30 cm depth, while the root length density was higher with underground drip irrigation below 30 cm^16^. Notably, the increase in root mass below 30 cm did increased the total dry matter on the ground.

Irrigation frequency also has a significant effect on root development. For example, the in zone for root uptake moved upward as the irrigation frequency of winter wheat increased^3^. Conversely, when irrigation frequency was reduced, bent grass developed a larger and deeper root system when irrigation frequency was reduced^17^. Researchers have performed experiments on the distribution and growth of sorghum roots in response to irrigation frequency. To reduce the effect of other factors on root growth, an experiment was conducted in a room with controlled temperature and artificial lighting. The results showed that root penetration depth was not limited, but root length density was proportionally lower in the upper profile of the less frequently irrigated treatments^18^.

Wheat is one of the world’s most important agricultural crops and is vital for food security^19^. Researchers have predicted that wheat yields must double by 2050 to meet future demands (FAO, 2009). Wheat is produced in many arid and semiarid regions around the world, including the Xinjiang Uyghur Autonomous Region in northwest China. Water shortages are a major constraint on wheat production in these areas. Irrigation is often needed to ensure high yields and stable production. The Food and Agriculture Organization of the United Nations estimates that nearly 8% of irrigated land uses sprinklers or microirrigation (FAO, 2009). Wheat is grown on 1.14 × 10^6^ ha in Xinjjiang Province. Two-thirds of this area is drip irrigated with water amounts ranging between 3000 and 7500 m^3^/ha. However, little is known about root growth and its relationship to water relations and the yield of drip-irrigated wheat in Xinjiang Province.

The objectives of these two years of field experiments were (1) to study the effect of irrigation frequency and irrigation amount on the root characteristics of drip-irrigated spring wheat (*Triticum aestivum* L.) and (2) to determine the relationship between these root characteristics and aboveground biomass and yield.

## Materials and methods

### Experimental site and experimental design

The field experiment was conducted during the 2013 and 2014 growing seasons at the Shihezi University Agronomy Experiment Station, Xinjiang Province, China (44° 26.5′ N, 86° 01′ E). The climate at the site climate was introduced in a previous paper^20^. The mean temperature during the wheat growing season was 20.6 °C during both growing seasons^20^. The precipitation amounts during the wheat growing seasons were 108 mm in 2013 and 78 mm in 2014 (Table 1). The soil type is gray desert soil according to the Chinese classification system and a Calcaric Fluvisol according to the FAO/UNESCO system (FAO, 1998). The physicochemical properties of the soil were similar in both years (Table 2).

**Table 1:** Monthly mean temperature and monthly rainfall at Shihezi from April 1 until harvest in 2013 and 2014.

	Mean temp (°C)		Rainfall (mm)	
	2013	2014	2013	2014
April	14.3	12.5	38.1	44.5
May	19.3	20.8	26.0	22.2
June	23.5	24.8	29.2	7.90
July	25.0	24.9	14.4	2.90

**Table 2:** Selected soil physical and chemical properties (0–60 cm depth) in the experiment plots.

Variable	Year	
	2013	2014
Clay (%)	21 ± 1.35	20 ± 1.05
Silt (%)	35 ± 2.42	33 ± 2.31
Sand (%)	44 ± 1.38	47 ± 1.56
pH	7.54 ± 0.54	7.21 ± 0.35
Organic matter (mg/kg)	24.6 ± 1.49	25.7 ± 1.03
Alkaline-N (mg/kg)	75.13 ± 3.57	71.39 ± 2.11
Olsen-P (mg/kg)	21.58 ± 2.01	22.64 ± 1.97
Available K (mg/kg)	157 ± 12	170 ± 14
Bulk density (g/cm^3^)	1.27 ± 0.27	1.15 ± 0.17
Saturated volumetric water content (%)	23.7 ± 0.5	22.9 ± 1.4

A split-plot design was used with the drip irrigation interval as the main plot factor and irrigation amount as the split-plot factor. The irrigation intervals were 13 d (D1), 10 d (D2), and 7 d (D3). The irrigation amounts were 3750 m^3^/ha (W1), 6000 m^3^/ha (W2), and 8250 m^3^/ha (W3). Every irrigation amount is shown in Table 3. The treatments were replicated three times.

**Table 3:** Shoot and root characteristics at flowering as affected by drip irrigation frequency and amount.

Year	Cultivar	Treatment		LAI	Root–shoot ratio	RDMPLA	Specific root length	SLA
		D	W					
2013	Xinchun 6	1	1	2.91 ± 0.23c	0.10 ± 0.002c	3.27 ± 0.31b	0.08 ± 0.01ab	186.00 ± 20.8a
			2	3.54 ± 0.19ab	0.10 ± 0.002c	1.98 ± 0.11de	0.08 ± 0.01a	191.13 ± 21.28a
			3	4.04 ± 0.15a	0.12 ± 0.002bc	1.50 ± 0.06ef	0.07 ± 0.01ab	165.35 ± 12.83ab
		2	1	2.81 ± 0.17c	0.11 ± 0.006bc	4.74 ± 0.32a	0.05 ± 0.01b	134.92 ± 14.61b
			2	3.93 ± 0.23ab	0.12 ± 0.03b	2.05 ± 0.11d	0.08 ± 0.01ab	146.57 ± 9.90ab
			3	4.10 ± 0.23a	0.15 ± 0.01a	2.00 ± 0.12d	0.05 ± 0.003b	162.62 ± 14.87ab
		3	1	3.39 ± 0.15bc	0.10 ± 0.002c	2.64 ± 0.12c	0.07 ± 0.001ab	149.90 ± 12.19ab
			2	4.01 ± 0.22a	0.10 ± 0.004c	1.40 ± 0.07 fg	0.08 ± 0.01ab	142.85 ± 13.64b
			3	4.04 ± 0.24a	0.10 ± 0.004c	0.94 ± 0.05 g	0.05 ± 0.001ab	142.87 ± 9.85b
			D	ns	ns	***	ns	*
			W	***	*	***	*	ns
			D * W	ns	ns	***	ns	ns
2014	Xinchun 6	1	1	2.91 ± 0.17f.	0.11 ± 0.01b	3.29 ± 0.22b	0.09 ± 0.01a	216.02 ± 16.10a
			2	3.48 ± 0.21e	0.11 ± 0.01b	1.99 ± 0.12c	0.07 ± 0.01abc	229.27 ± 23.52a
			3	4.48 ± 0.30c	0.11 ± 0.002b	1.44 ± 0.09de	0.06 ± 0.003bcd	241.44 ± 14.86a
		2	1	3.95 ± 0.31de	0.12 ± 0.01b	3.90 ± 0.41a	0.06 ± 0.01bcd	256.02 ± 25.37a
			2	5.04 ± 0.13ab	0.16 ± 0.004a	1.60 ± 0.04 cd	0.07 ± 0.01abcd	258.23 ± 23.07a
			3	4.66 ± 0.08bc	0.19 ± 0.03a	1.78 ± 0.03 cd	0.04 ± 0.01d	270.13 ± 17.41a
		3	1	4.40 ± 0.08 cd	0.12 ± 0.001b	2.05 ± 0.04c	0.08 ± 0.01ab	234.40 ± 18.39a
			2	5.11 ± 0.09ab	0.11 ± 0.002b	1.07 ± 0.02e	0.06 ± 0.01abcd	237.87 ± 21.47a
			3	5.50 ± 0.10a	0.11 ± 0.01b	0.69 ± 0.13f.	0.04 ± 0.01 cd	274.85 ± 32.51a
			D	***	*	***	ns	ns
			W	***	*	***	**	ns
			D * W	**	ns	**	ns	ns
	Xinchun 22	1	1	2.59 ± 0.19e	0.13 ± 0.001b	2.69 ± 0.17b	0.09 ± 0.02ab	199.35 ± 19.15bc
			2	3.03 ± 0.12de	0.14 ± 0.02b	1.86 ± 0.08 cd	0.1 ± 0.02a	214.09 ± 13.85bc
			3	3.75 ± 0.22bc	0.12 ± 0.001b	1.41 ± 0.08d	0.09 ± 0.01a	192.03 ± 18.19c
		2	1	3.26 ± 0.18 cd	0.13 ± 0.03b	3.25 ± 0.18a	0.06 ± 0.01ab	229.95 ± 22.25bc
			2	4.30 ± 0.11a	0.13 ± 0.01b	2.20 ± 0.06c	0.06 ± 0.003ab	216.69 ± 20.09bc
			3	4.01 ± 0.14ab	0.21 ± 0.01a	1.89 ± 0.07c	0.06 ± 0.005ab	276.16 ± 31.32b
		3	1	3.44 ± 0.32 cd	0.12 ± 0.002b	2.67 ± 0.26b	0.05 ± 0.004b	193.99 ± 20.29c
			2	4.21 ± 0.11ab	0.14 ± 0.01b	1.99 ± 0.06c	0.05 ± 0.01b	246.32 ± 29.57bc
			3	4.38 ± 0.18a	0.13 ± 0.004b	1.73 ± 0.07d	0.05 ± 0.003b	358.01 ± 38.42a
			D	***	ns	***	**	*
			W	***	ns	***	ns	*
			D * W	ns	*	ns	ns	*

Abbreviations: *LAI* leaf area index, *RDMPLA* root dry weight per unit leaf area, *SLA* specific leaf area, *ns* represents no significance at the 0.05 probability level.

*Represents significance at the 0.05 probability level.

**Represents significance at the 0.01 probability level.

The spring wheat (*Triticum aestivum* L.) cultivar Xinchun 6 used in both 2013 and 2014. This cultivar was chosen because it performed well under drip irrigation in preliminary tests. A second spring wheat cultivar, Xinchun 22, was included in the experiment in 2014. The seeding rate of both cultivars was 618 kg/ha. Preliminary studies indicated that this rate resulted in the highest yield of drip-irrigated spring wheat at the experimental site.

**Figure 1:**
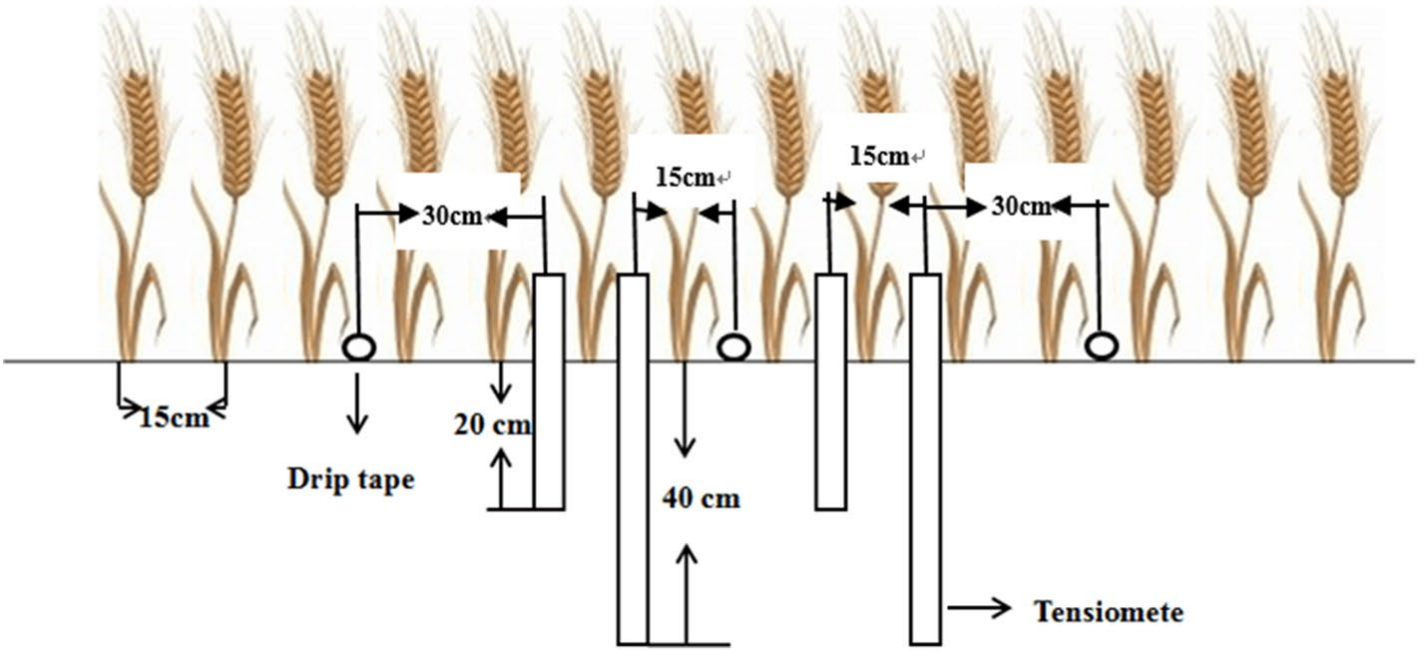
Sketch map showing the relative positions of the drip laterals, the wheat rows, and the Watermark tensiometers.

The area of each main plot was 18.9 m^2^. The area of each split-plot was 6.3 m^2^. There were 14 rows of wheat in each split-plot. The rows were 3 m long, and the intrarow spacing was 0.15 m. There were two wheat rows on each side of every drip tape (Fig. 1, the spacing between the drip tapes was 60 cm). There was a 30 cm space between each emitter. The emitter discharge rate was 3.20 l/h. Water movement between the plots was prevented by burying waterproof membranes to a depth of 80 cm below the soil surface between each plot. The plots were irrigated after wheat emerged (April 10, 2013 and April 6, 2014).

The plots were fertilized with 600 kg/ha urea and 225 kg/ha diammonium phosphate [(NH_4_)_2_HPO_4_]. Twenty-five percent of the urea (i.e., 150 kg/ha) and all of the P fertilizer were applied at sowing. The remainder of the urea was applied at the following times: 15% at the three-leaf stage, 30% at jointing, 30% at booting stage, 15% at flowering, and 10% at grain filling^20^.

### Soil water potential

Four tensiometers (Irrometer Company, Riverside, CA, USA) were installed in each split plot to monitor soil water potential. Two tensiometers were installed 15 cm from the drip tape, one at the 0–20 cm depth and one at the 20–40 cm depth. The other two tensiometers were installed 30 cm from the drip tape, also at depths of 0–20 and 20–40 cm. Soil moisture was measured just before and after each irrigation event (Fig. 1).

### Root weight density (RWD) and root length density (RLD)

Root morphology was assessed at flowering during 2013 and 2014 using the core sampling method^21^. Three hills were collected from the 0–40 cm depth of each split plot using a soil corer (5 cm diam). The samples were divided into 0–20 and 20–40 cm depths and then washed with tap water on a 0.5 mm mesh screen. Grass roots and other organic debris were removed from the samples. The roots were then digitally analyzed using a flatbed image scanner (Epson V500; Epson America, Inc., San Jose, CA, USA) as described by Kato et al.^22^. The root lengths were determined with WinRHIZO commercial software (Regent Instruments, Montreal, QC, Canada). After scanning, the samples were dried in an oven at 75 °C for 72 h and then weighed. Using data from the above measurements, we calculated root length density (RLD, root length per volume of soil, cm/cm^3^), root weight density (RWD, root weight per volume of soil, g/m^3^) and specific root length (the ratio of root length to root weight, m/g).

### Leaf area index at flowering (LAImax), dry matter, and grain yield

The aboveground plant samples were collected along with roots at flowering. The samples were separated into leaves, stems, and panicles. The green leaf area was measured with an LI-3100 leaf area meter (LI-COR Inc., Lincoln, NE, USA). The samples were dried in an oven at 75 °C for at least 72 h and then weighed. Specific leaf area (SLA) was calculated by dividing leaf weight by leaf area^23^. The root:shoot ratio (dry weight basis) and the root dry weight per unit leaf area (RDMPLA) were also calculated. Grain yield was determined from a 1 m^2^ area in each split plot and adjusted to a moisture content of 0.14 g H_2_O/g fresh weight. Panicle dry weight and yield components were determined using three subsamples from three 1 m^2^ areas in each split plot.

### Net photosynthetic rate (Pn)

The Pn of five flag leaves in each subplot was measured using a LI-6400 portable open-flow gas exchange system (Li-COR Inc., NE, USA) under 1800 μmol/m^2^/s light intensity from a red/blue LED light source between 10:30 and 12:30 h on three consecutive days during flowering^20^.

### Crop evapotranspiration (ETc) and water use efficiency (WUE)

Crop evapotranspiration (ETc) was determined using the soil water balance method described in our previous paper^20^. Water use efficiency (WUE) was calculated by dividing grain yield by ETc^20^.

### Statistical analysis

The data were analyzed using the generalized linear model (GLM) procedure (SPSS 16.0). Differences between means were compared using Fisher’s least significant difference (LSD) tests at the 5% probability level^20^.

## Results

### Soil water potential

**Figure 2:**
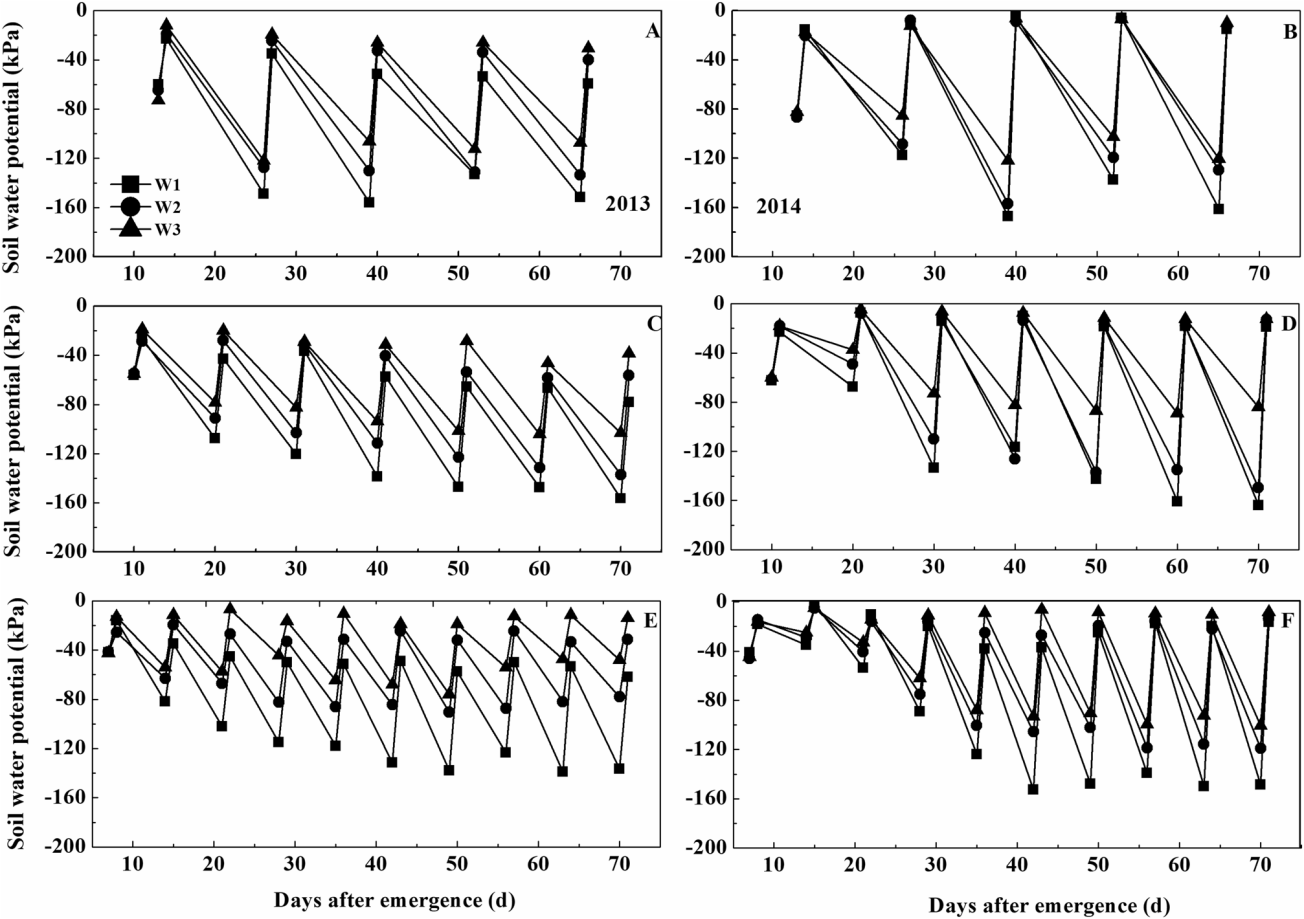
Temporal changes in soil water potential in the 0–20 cm depth under ‘Xinchun 6’. (**A**) Soil water potential under D1 treatment in 2013. (**B**) Soil water potential under D1 treatment in 2014. (**C**) Soil water potential under D2 treatment in 2013. (**D**) Soil water potential under D2 treatment in 2014. (**E**) Soil water potential under D3 treatment in 2013. (**F**) Soil water potential under D3 treatment in 2014.

**Figure 3:**
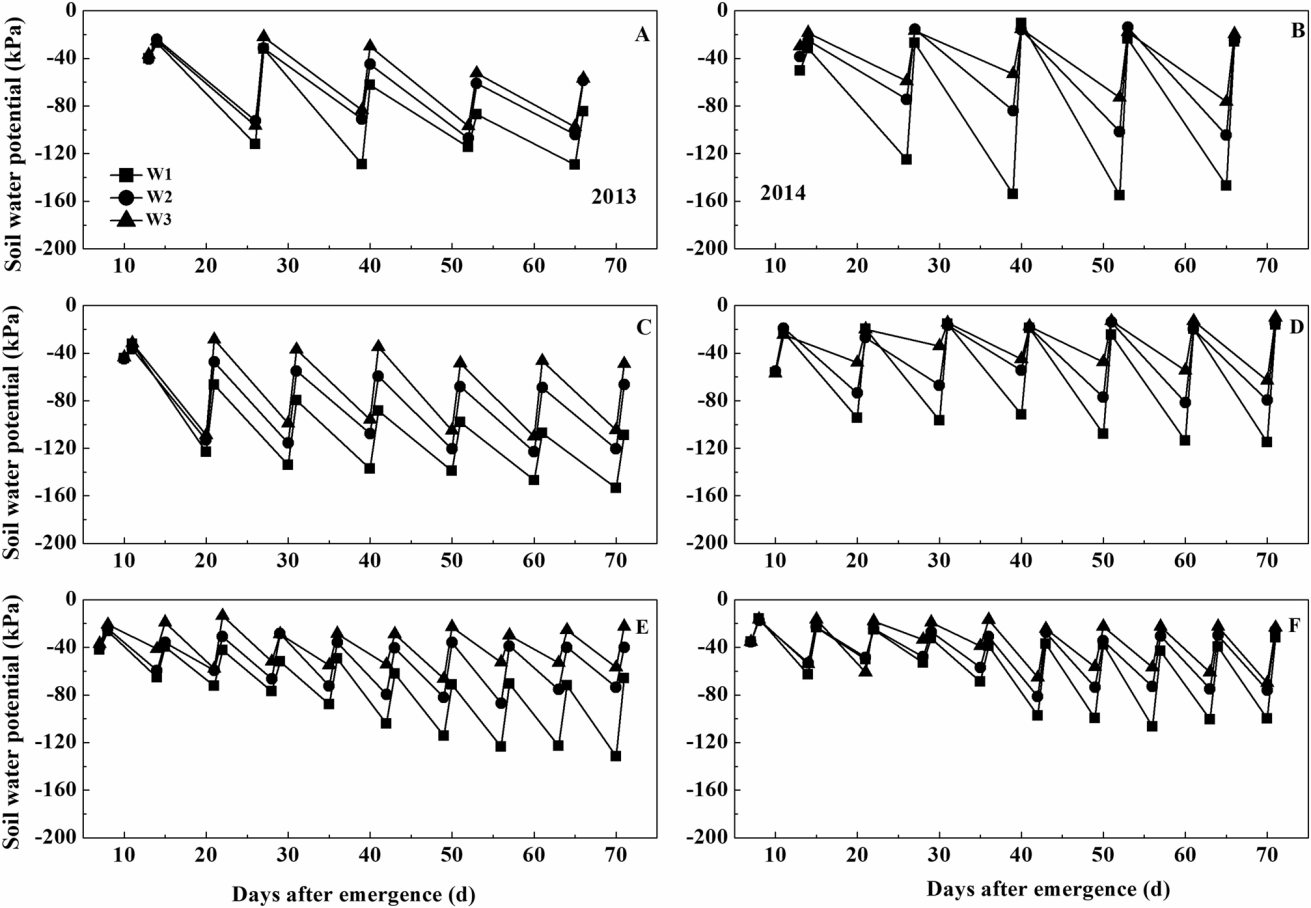
Temporal changes in soil water potential in 20–40 cm depth under ‘Xinchun 6’. (**A**) Soil water potential under D1 treatment in 2013. (**B**) Soil water potential under D1 treatment in 2014. (**C**) Soil water potential under D2 treatment in 2013. (**D**) Soil water potential under D2 treatment in 2014. (**E**) Soil water potential under D3 treatment in 2013. (**F**) Soil water potential under D3 treatment in 2014.

Soil water potentials decreased as the soil dried between irrigation events and then increased to near their original level after irrigation. The fluctuations were greater at depths of 0–20 cm than at depths of 20–40 cm (Figs. 2, 3).

At the 0–20 cm depth, there were differences among irrigation amounts and irrigation frequency before every irrigation event. The D3 plot had the highest water potentials than the D2 and D1 treatments under the W2 and W3 treatments, and the irrigation frequency was not different under the W1 treatment. Compared with the different irrigation amounts before irrigation, the soil water potentials decreased in the order W3 > W2 > W1.

At depths of 0–20 cm, the maximum water potentials ranged between − 5 and − 40 kPa. The maximum soil water potentials were generally not affected by either irrigation frequency or irrigation amount; the exceptions were in the D2 and D3 plots in 2013 (Fig. 2). The minimum soil water potentials ranged between − 80 and − 160 kPa. Minimum soil water potentials decreased in the order W3 > W2 > W1. The differences in minimum water potential among W1, W2, and W3 were larger in the D2 and D3 plots than in the D1 plots.

The maximum water potentials in the 20–40 cm depth ranged between − 20 and − 100 kPa (Fig. 3). These values were slightly lower than those at depths of 0–20 cm (Figs. 2, 3). The maximum soil water potentials in the 20–40 cm depth were similar in the D1, D2, and D3 plots (Fig. 3). There were some differences in maximum soil water among W1, W2, and W3 in 2013 but not in 2014. The minimum soil water potentials ranged between − 40 and − 160 kPa. The minimum water potentials decreased in the order W3 > W 2 > W1, with the largest differences being in the D1 plots in 2014.

At 20–40 cm depth, we observed that the D3 plot had the highest water potentials compared with the D2 and D1 treatments. Compared with the different irrigation amounts before irrigation, the soil water potentials decreased in the order W3 > W2 > W1.D3W3 had the highest water potential before every irrigation in all treatments.

### Root characteristics

**Figure 4:**
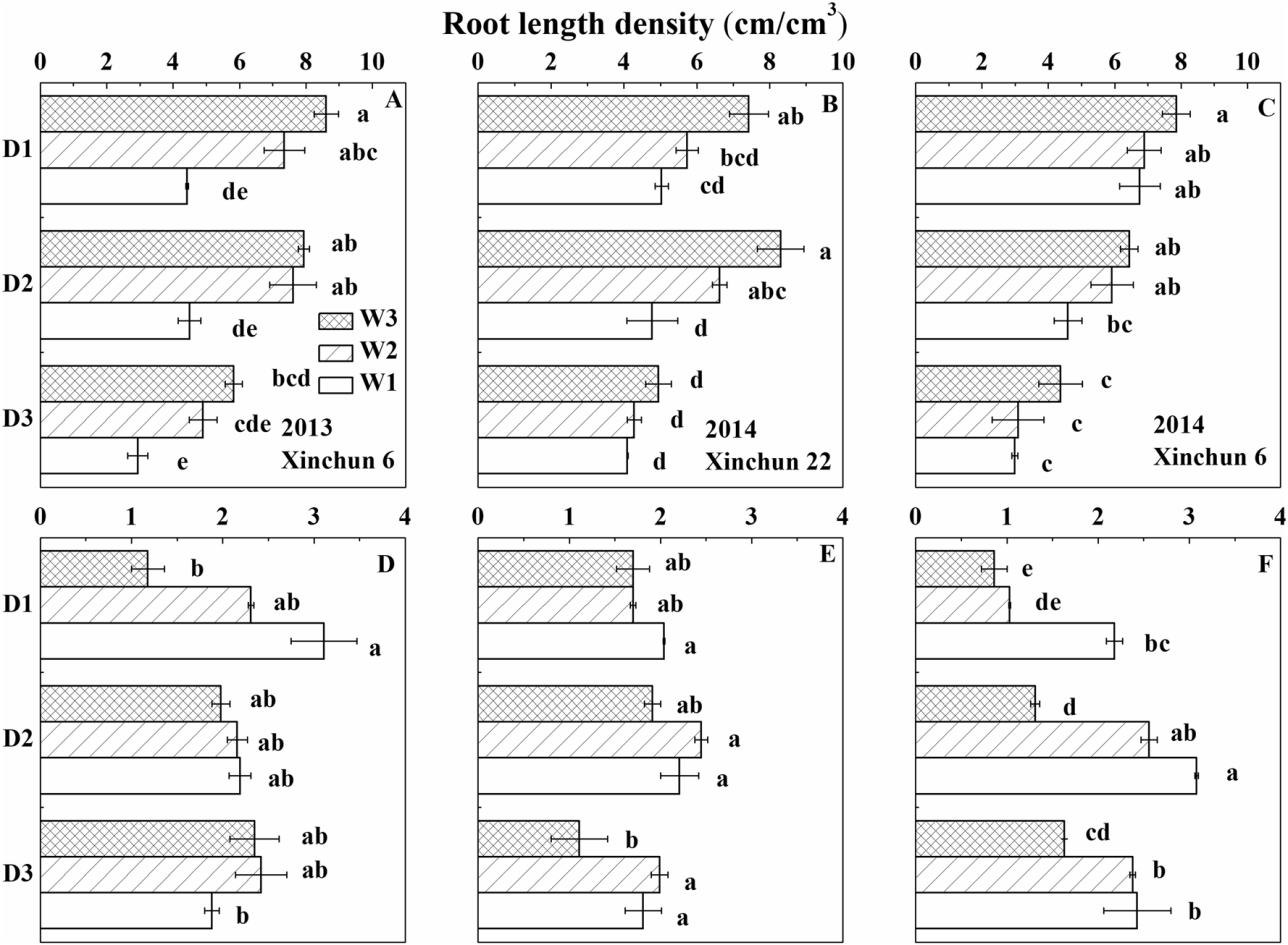
Root length density at flowering as affected by irrigation frequency and amount. (**A**) The root length density of ‘Xinchun 6’ in the 0–20 cm depths in 2013. (**B**) The root length density of ‘Xinchun 22’ in the 0–20 cm depths in 2014. (**C**) The root length density of ‘Xinchun 6’ in the 0–20 cm depths in 2014. (**D**) The root length density of ‘Xinchun 6’ in the 20–40 cm depths in 2013. (**E**) The root length density of ‘Xinchun 22’ in the 20–40 cm depths in 2014. (**F**) The root length density of ‘Xinchun 6’ in the 20–40 cm depths in 2014. Error bars represent ± SE (n = 10). Bars within a panel with a different letter are significantly different at *P* < 0.05.

The root length density in the 0–20 cm depth ranged between 2.99 and 9.13 cm/cm^3^ (Fig. 4). The RLD of the two cultivars was significantly affected by irrigation frequency and irrigation amount in the two years. The RLD in the D1 plots was 2–27% greater than that in the D2 plots and 49–106% greater than that in the D3 plots of Xinchun 6. The RLD was greatest in D1W3 and least in D3W1. The RLD decreased in the order W3 > W2 > W1. The RLD in the D2 plots was 8% greater than that in the D1 plots and 48% greater than that in the D3 plots of Xinchun 22. The RLD of Xinchun 22 was greatest in D2W3 and least in D3W1. The RLD generally decreased in the order W3 > W2 > W1.

The root length density in the 20–40 cm depth ranged between 0.86 and 3.11 cm/cm^3^ (Fig. 4). The RLD of the two cultivars was significantly affected by irrigation frequency and irrigation amount in the two years. The RLD in the D1 plots was 21.5–25.1% greater than that in the D2 plots and 59.5–33.5% greater than that in the D3 plots of the two cultivars. The RLD decreased in the order W1 > W2 > W3. The RLD was greatest in D1W1 and least in D3W3.

**Figure 5:**
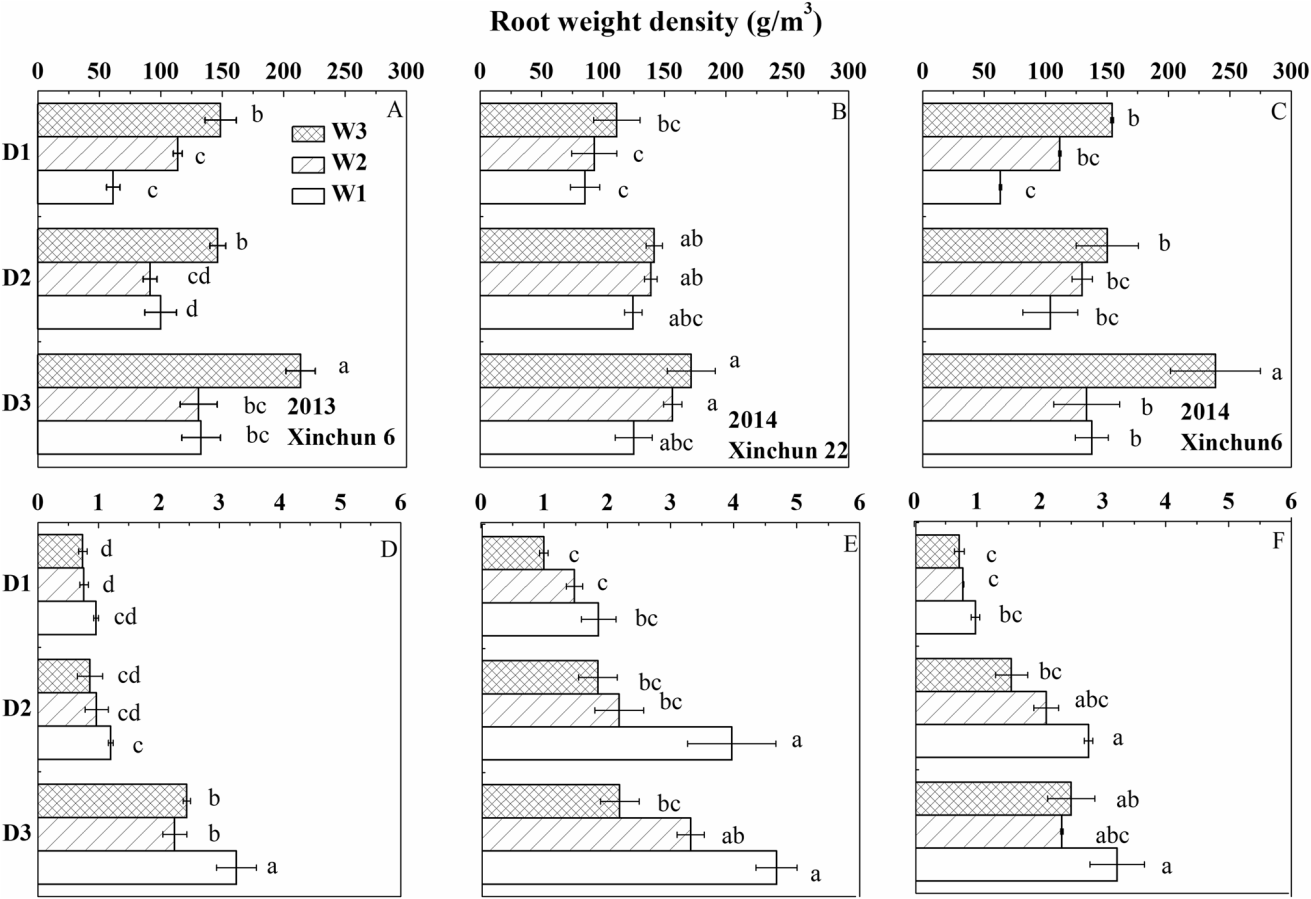
Root weight density at flowering as affected by irrigation frequency and amount. (**A**) The root weight density of ‘Xinchun 6’ in the 0–20 cm depths in 2013. (**B**) The root weight density of ‘Xinchun 22’ in the 0–20 cm depths in 2014. (**C**) The root weight density of ‘Xinchun 6’ in the 0–20 cm depths in 2014. (**D**) The root weight density of ‘Xinchun 6’ in the 20–40 cm depths in 2013. (**E**) The root weight density of ‘Xinchun 22’ in the 20–40 cm depths in 2014. (**F**) The root weight density of ‘Xinchun 6’ in the 20–40 cm depths in 2014. Error bars represent ± SE (n = 10). Bars within a panel with a different letter are significantly different at *P* < 0.05.

The root weight density ranged between 61.3 and 238.4 g/m^3^ at depths of 0–20 cm (Fig. 5). The RWDs of the two cultivars were significantly affected by irrigation frequency and irrigation amount over the two years. The RWD in the D3 plots was 36.7–11.9% greater than that in the D2 plots and 51.1–56.7% greater than that in the D1 plots of the two cultivars. The RWD decreased in the order W3 > W2 > W1. The RWD was greatest in D3W3 and least in D1W1.

At 20–40 cm depth, the RWD ranged between 0.70 and 4.73 g/m^3^. The RWD in the D3 plots was 70.3–27.5% greater than that in the D2 plots and 229.7–137.1% greater than that in the D1 plots of the two cultivars. The RWD of both cultivars was greatest in D3W1 and lowest in D1W3. The RWD decreased in the order W1 > W2 > W3.

Irrigation amount, but not irrigation frequency, had significant effects on the specific root length of Xinchun 6 in both years (Table 3). In 2013, the specific root length was greatest in W2. In 2014, the specific root length was greatest in both W1 and W2. In contrast to Xinchun 6, irrigation frequency significantly affected the specific root length of Xinchun 22. The specific root length in the D1 plots was 26.7–42.6% greater than that in the D2 plots and 20.6–60.2% greater than that in the D3 plots.

### Aboveground biomass

**Figure 6:**
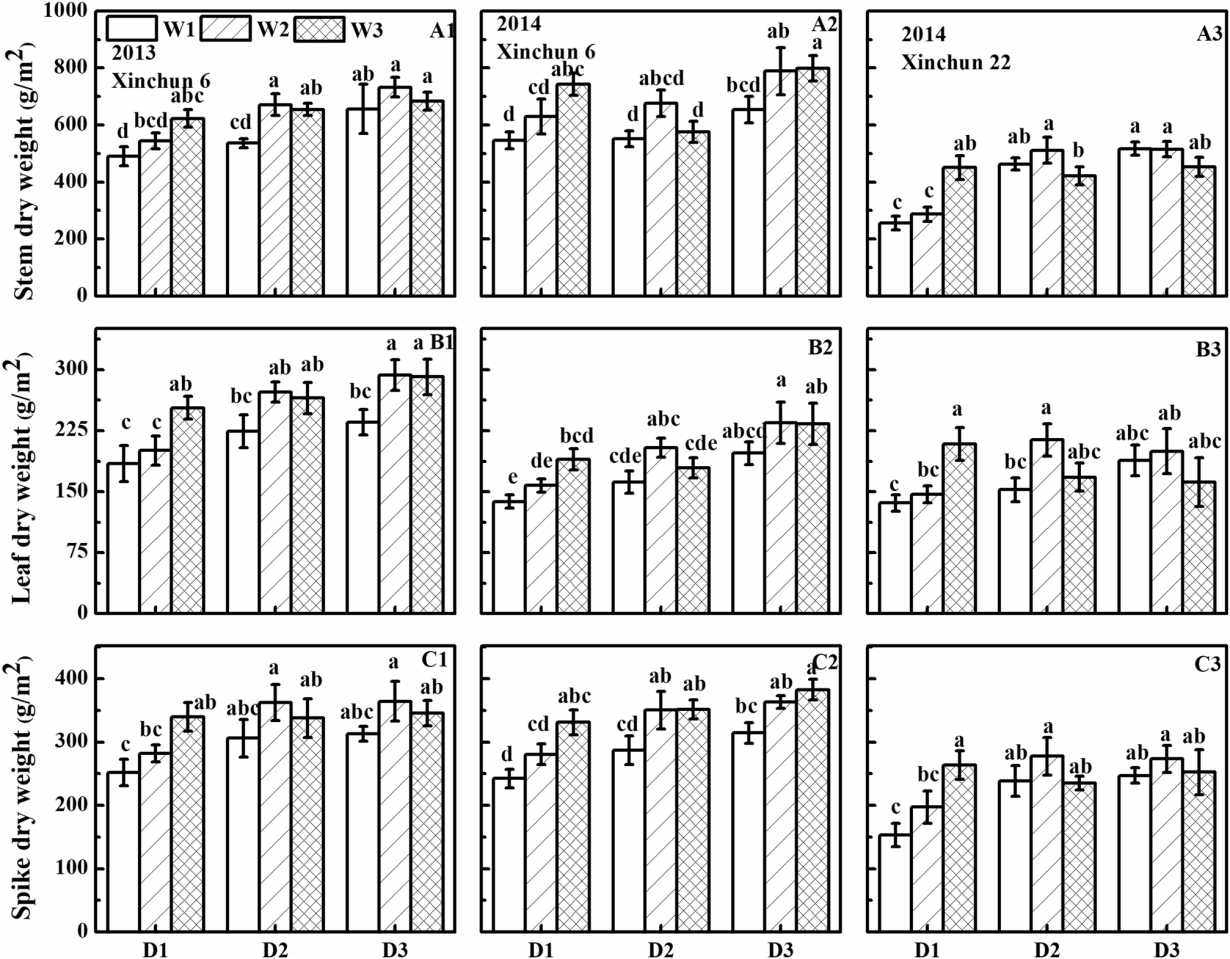
Dry weight of different wheat organs at flowering as affected by irrigation frequency and amount. (**A1**) The stem dry matter of ‘Xinchun 6’ in 2013. (**B1**) The leaf dry matter of ‘Xinchun 6’ in 2013. (**C1**) The spike dry matter of ‘Xinchun 6’ in 2013. (**A2**) The stem dry matter of ‘Xinchun 6’ in 2014. (**B2**) The leaf dry matter of ‘Xinchun 6’ in 2014. (**C2**) The spike dry matter of ‘Xinchun 6’ in 2014. (**A3**) The stem dry matter of ‘Xinchun 22’ in 2014. (**B3**) The leaf dry matter of ‘Xinchun 22’ in 2014. (**C3**) The spike dry matter of ‘Xinchun 22’ in 2014. Error bars represent ± SE (n = 10). Bars within a panel with a different letter are significantly different at *P* < 0.05.

Irrigation frequency and irrigation amount significantly affected the stem, leaf, and spike dry weights of Xinchun 6 (Fig. 6). The dry weights were generally greatest in the D3 plots and least in the D1 plots. The exception was that stem dry weights in the D1 plots were greater than those in the D2 plots in 2014. The dry weights generally decreased in the order W3 > W2 > W1 in the D1 plots (Fig. 6). In the D2 and D3 plots, stem, leaf, and spike dry weights were greater in W2 than in W3. Irrigation frequency, but not irrigation amount, significantly affected the stem, leaf, and spike dry weights of Xinchun 22. The dry weights were generally greatest in the D3 plots and least in the D1 plots.

### Photosynthesis and LAI

**Figure 7:**
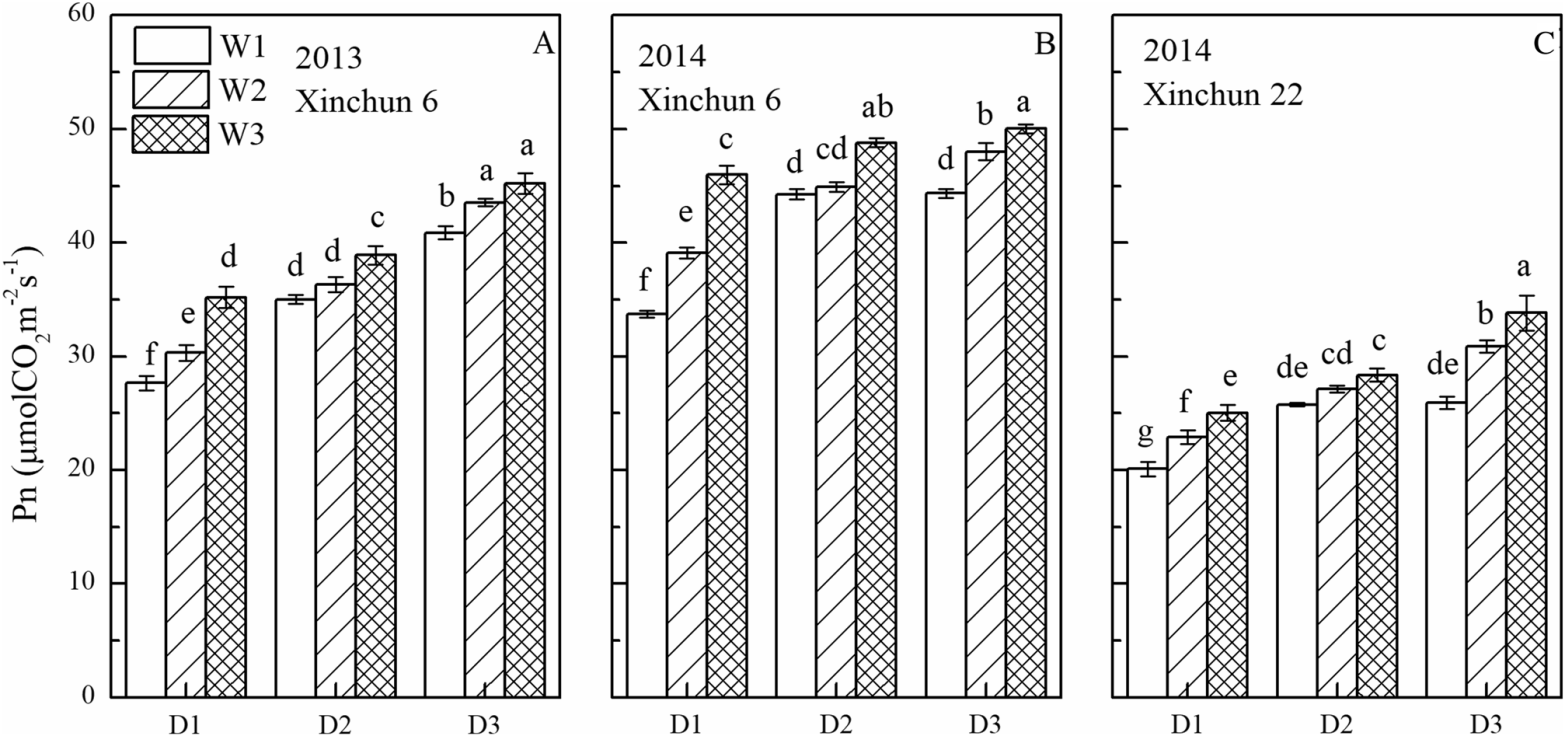
Leaf net photosynthetic rate (Pn) at early grain filling stage as affected by irrigation frequency and amount. (**A**) Pn of Xinchun 6 in 2013. (**B**) Pn of Xinchun 6 in 2014. (**C**) Pn of Xinchun 22 in 2014. Leaf Pn was calculated across five replicates for each day and then averaged for three days. Error bars represent ± SE (n = 10). Bars within a frame with a different letter are significantly different at *P* < 0.05.

Leaf Pn was significantly affected by irrigation frequency, irrigation amount, and their interaction (Fig. 7). The leaf Pn in the D3 plots was 17.6–6.30% greater than that in the D2 plots and 39.1–24.7% greater than that in the D1 plots. Leaf Pn decreased in the order W3 > W2 > W1, with W3 being 8.30–8.93% greater than that in W2 and 15.2–19.6% greater than that in W1. For both cultivars, leaf Pn was greatest in D3W3 and least in D1W1.

The LAI was significantly affected by irrigation frequency in 2014 but not in 2013 (Table 3). In 2014, the LAI in the D3 plots was 9.89–3.89% greater than that in the D2 plots and 38.1–28.5% greater than that in the D1 plots. Irrigation amount significantly affected LAI in both years. The LAI decreased in the order W3 > W2 > W1, with W3 being 6.01–6.19% greater than that in W2 and 33.6–30.0% greater than that in W1. In 2014, LAI was always greatest in the D3W3 treatment and least in the D1W1 treatment. The LAI of Xinchun 6 was 20% greater than that of Xinchun 22. The change of LAI was similar with leaf Pn.

The SLA of Xinchun 6 was significantly affected by irrigation frequency in 2013 but not in 2014. In 2013, the SLA in the D1 plots was 22.1% greater than that in the D2 plots and 24.5% greater than that in the D3 plots. The irrigation amount and interaction effects on the SLA of Xinchun 6 were not significant in either year. Irrigation frequency, irrigation amount, and their interaction had significant effects on the SLA of Xinchun 22. The SLA in the D3 plots was 10.5% greater than that in the D2 plots and 31.9% greater than that in the D1 plots. The SLA decreased in the order W3 > W2 > W1, with that in W3 being 32.6% greater than that in W2 and 22.0% greater than that in W1.

### Relationship between grain yield and root characteristic

The root/shoot ratio of Xinchun 6 was significantly affected by irrigation frequency in 2013, being 15.6–16.7% greater in the D2 plots than in the D1 and D3 plots. The irrigation amount significantly affected the root/shoot ratio of Xinchun 6 in 2014. The root/shoot ratio was 8.54–7.87% greater in W3 than in W1 and W2. In both cultivars, D2W3 had the greatest root/shoot ratio. Irrigation frequency and irrigation amount both had significant effects on RLDPMA. The RLDPMA was 20.4–55.7% greater in D2 than in D1 and D3. The RLDPMA generally decreased in the order W1 > W2 > W3. In both cultivars, D2W1 had the highest RLDPMA (Table 3).

**Figure 8:**
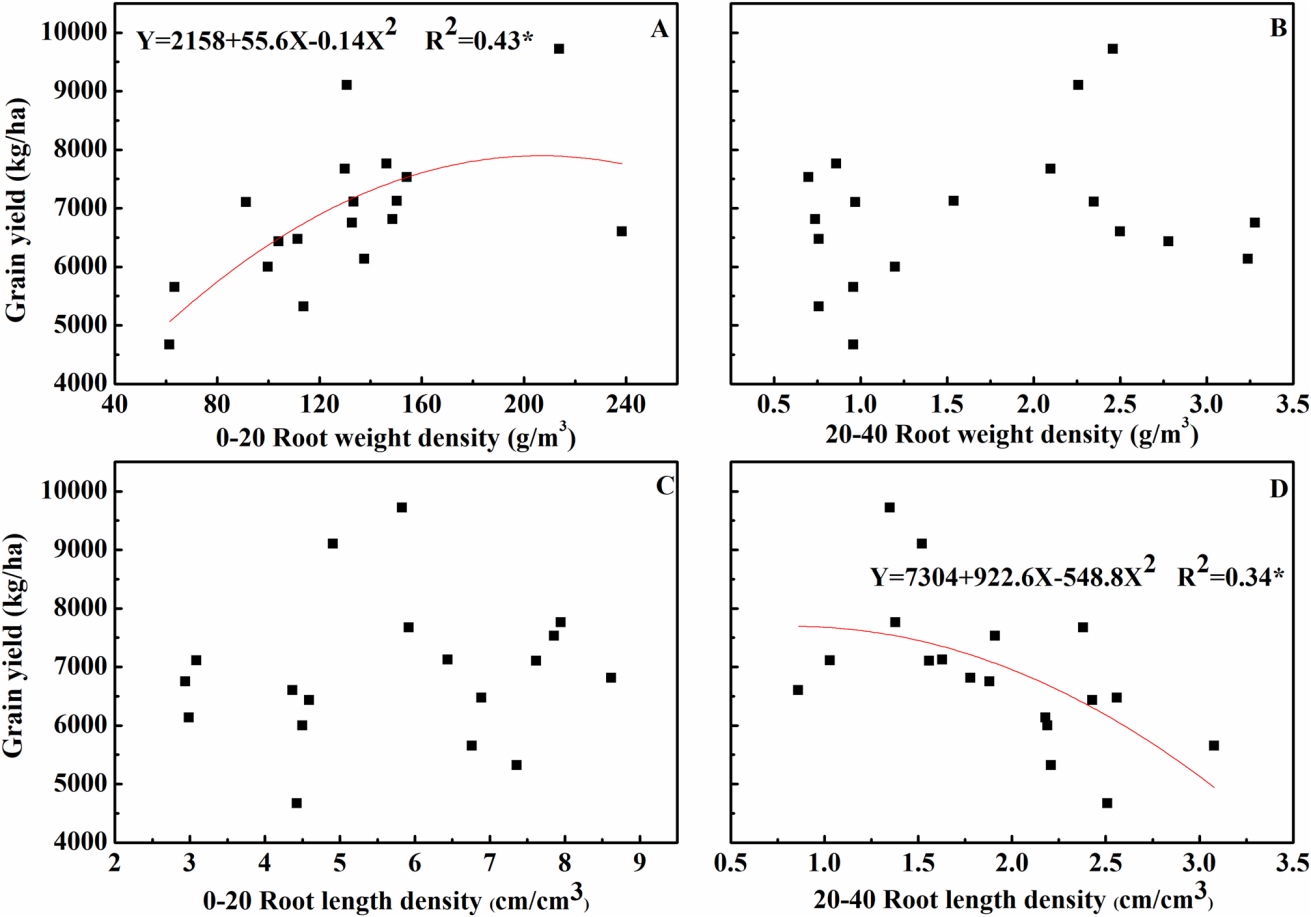
Relationship between the grain yield of Xinchun 6 and root characteristic. (**A**) Relationship between root weight density in 0–20 cm depth and grain yield. (**B**) Relationship between root weight density in 20–40 cm depth and grain yield. (**C**) Relationship between root length density in 0–20 cm depth and grain yield. (**D**) Relationship between root weight density in 20–40 cm depth and grain yield.

Regression analysis indicated a significant relationship between RWD at the 0–20 cm depth and grain yield (R^2^ = 0.43) of Xinchun 6. In contrast, grain yield increased as RWD at the 0–20 cm depth increased. Regression analysis also indicated a significant relationship between the RLD at 20–40 cm depth and the grain yield (R^2^ = 0.34) of Xinchun 6, and the grain yield increased as the RLD at 20–40 cm depth decreased. The relationship was best described by a binomial function (Fig. 8).

### Grain yield and its components

Irrigation frequency significantly affected the panicle number of both cultivars in both years (Table 4). Averaged across irrigation amounts, panicle numbers in the D3 plots were 4–38% greater than in the D1 plots and 11–14% greater than in the D2 plots. Irrigation amount also had significant effects on panicle number. In Xinchun 6, panicle numbers in W3 were 36.5–19.7% greater than those in W1 and 25.5–6.09% greater than those in W2. In Xinchun 22, panicle numbers in W2 were 5.9% greater than those in W1 and 42.5% greater than those in W2.

**Table 4:** Wheat grain yield and yield components as affected by drip irrigation frequency and amount.

Year	Cultivar	Treatment		Effective panicles (no./m^2^)	Spikelets per panicle	1000 grain weight (g)	Grain yield (kg/ha)
		D	W				
2013	Xinchun 6	1	1	303 ± 7.81d	24.8 ± 0.940c	52.2 ± 1.46a	5670 ± 187c
			2	347 ± 17.7d	28.1 ± 1.60bc	46.9 ± 2.07b	6320 ± 385c
			3	535 ± 33.7b	26.6 ± 1.01c	40.7 ± 1.58e	7310 ± 508b
		2	1	439 ± 11.7c	24.9 ± 1.35c	45.8 ± 1.03bc	6000 ± 369c
			2	456 ± 11.51c	31.8 ± 1.94ab	42.2 ± 1.76cde	7100 ± 447b
			3	541 ± 25.0b	28.6 ± 1.28bc	42.5 ± 0.60cde	7760 ± 539b
		3	1	490 ± 13.3bc	25.7 ± 1.37c	44.4 ± 0.79bcd	6750 ± 443bc
			2	537 ± 19.9b	35.2 ± 1.61a	42.6 ± 0.81cde	9100 ± 450a
			3	607 ± 26.1a	34.0 ± 1.07a	41.3 ± 0.98de	9720 ± 472a
			D	***	***	**	**
			W	***	***	***	**
			D * W	**	ns	**	ns
2014	Xinchun 6	1	1	437 ± 11.3e	22.8 ± 2.12c	51.6 ± 1.55a	5650 ± 462c
			2	523 ± 16.27d	24.3 ± 2.14c	48.2 ± 2.54ab	6470 ± 539abc
			3	552 ± 11.6d	26.4 ± 3.18bc	43.8 ± 1.73bc	7530 ± 409ab
		2	1	568 ± 10.9c	26.9 ± 1.70bc	45.6 ± 1.16bc	6430 ± 472abc
			2	616 ± 26.4b	35.0 ± 1.97a	47.5 ± 3.27abc	7670 ± 376a
			3	688 ± 21.7a	23.3 ± 1.41c	42.4 ± 2.12 cd	7120 ± 244a
		3	1	568 ± 20.8c	22.2 ± 1.74c	46.2 ± 1.93abc	6130 ± 140bc
			2	635 ± 23.5b	30.9 ± 2.76ab	35.0 ± 1.93e	7110 ± 899ab
			3	640 ± 24.3b	27.0 ± 2.25bc	37.5 ± 1.16de	6600 ± 802ab
			D	***	ns	***	ns
			W	***	**	***	*
			D * W	**	*	*	ns
	Xinchun 22	1	1	674 ± 9.68 fg	29.9 ± 2.21abc	31.4 ± 3.44ab	5680 ± 286bcd
			2	740 ± 4.53c	30.2 ± 1.93abc	30.7 ± 2.32ab	6410 ± 305abc
			3	766 ± 1.80b	36.1 ± 3.00a	31.9 ± 2.40ab	7330 ± 311a
		2	1	661 ± 7.87 g	26.2 ± 2.10bc	29.8 ± 2.32ab	5140 ± 570 cd
			2	684 ± 4.80ef	33.2 ± 3.18a	33.6 ± 1.71a	6960 ± 513ab
			3	700 ± 6.67de	30.9 ± 2.40abc	29.4 ± 2.34ab	5870 ± 407bcd
		3	1	707 ± 3.60d	24.6 ± 1.60c	26.4 ± 2.50b	5050 ± 220d
			2	739 ± 8.72c	32.3 ± 2.49ab	27.1 ± 1.72ab	6050 ± 558abcd
			3	819 ± 3.33a	24.9 ± 1.61c	26.4 ± 2.52b	5520 ± 764 cd
			D	***	*	*	ns
			W	***	*	ns	**
			D * W	***	ns	ns	ns

*ns* represents no significance at the 0.05 probability level.

*Represents significance at the 0.05 probability level.

**Represents significance at the 0.01 probability level.

Irrigation frequency significantly affected the spikelet number of Xinchun 6 in 2013 and Xinchun 22 in 2014. Irrigation amount significantly affected the spikelet number of both cultivars in both years. Spikelet numbers were generally greatest in W2. The exception was that in the D1 plots in 2014, W3 had the greatest spikelet number.

The 1000 grain weight was significantly affected by irrigation frequency in both years. Averaged across irrigation amounts, the 1000 grain weight in the D1 plots was 5.01% greater than that in the D2 plots and 15.4% greater than that in the D3 plots. The irrigation amount significantly affected the 1000 grain weight of Xinchun 6 but not Xinchun 22. The 1000 grain weights tended to be highest in W1. The 1000 grain weights were similar in W2 and W3.

Grain yield was significantly affected by irrigation frequency in 2013. Averaged across irrigation amounts, the yield in the D3 plots was 3.29% greater than that in the D2 plots and 32.5% greater than that in the D1 plots. Irrigation amount had significant effects on yield in both years. In 2013, grain yields decreased in the order W3 > W2 > W1. In 2014, grain yield decreased in the order W3 > W2 > W1 in the D1 plots. In the D2 and D3 plots, grain yields decreased in the order W2 > W3 > W1.

### WUE and ETc

The WUE was significantly affected by irrigation frequency and irrigation amount in 2013 but only by irrigation amount in 2014 (Table 5). The WUE in D3 was 8.33% greater than that in D2 and 28.4% greater than that in D1. The WUE in W1 was 14.9% greater than that in W2 and 66.2% greater than that in W3. The WUE was greatest in D3W1 in 2013 and D2W1 in 2014. The WUE was lowest in D1W3 in 2013 and D3W3 in 2014.

**Table 5:** Water use efficiency (WUE) and crop evapotranspiration (ETc) of spring wheat cultivar ‘Xinchun 6’ as affected by irrigation frequency and amount.

Year	Treatment		WUE (kg/m^3^)	ETc (mm)	Year	Treatment		WUE (kg/m^3^)	ETc (mm)
	D	W				D	W		
2013	1	1	0.82a	566c	2014	1	1	1.03a	546c
		2	0.70b	764b			2	0.85ab	762b
		3	0.69b	982a			3	0.79b	955a
	2	1	1.09a	548c		2	1	1.18a	543a
		2	0.93ab	767b			2	1.01a	761b
		3	0.79b	984a			3	0.73b	971a
	3	1	1.24a	543c		3	1	1.14a	539c
		2	1.21ab	748b			2	0.94a	755b
		3	1.01b	960a			3	0.68b	976a
		D	*	ns			D	ns	ns
		W	**	**			W	**	**
		D * W	ns	ns			D * W	ns	ns

*ns* represents no significance at the 0.05 probability level.

*Represents significance at the 0.05 probability level.

**Represents significance at the 0.01 probability level.

The ETc was similar in 2013 and 2014 (Table 5). The irrigation amount significantly affected ETc, but the irrigation frequency and interaction effects were not significant. Averaged across years and irrigation frequency treatments, the ETc in W1 was 77.4% less than that in W2 and 27.9% less than that in W3.

## Discussion

The soil water potential at different soil depths is directly affected by the irrigation method^24^. In our study, the soil water potentials at both 0–20 and 20–40 cm decreased rapidly after irrigation and then returned to near their original level after irrigation (Figs. 2, 3). This indicated that all of the treatment combinations met the water demands of the wheat.

Plant growth depends largely on the absorption and utilization of soil water. The importance of plant roots as suppliers of water and minerals has been discussed by many researchers^25–28^. Root system architecture is affected by temporal changes in the soil environment, including soil moisture^29–31^. Compared with flood irrigation practices, drip irrigation increases root growth in the surface soil. A previous report indicated that high-frequency drip irrigation restricted cotton root growth to the upper 30–40 cm depth of the soil profile^32^. Therefore, only the 0–40 cm depth was considered in this study.

Researchers commonly use RLD and RWD to characterize root systems^33^. Both the RLD and RWD were greater at the 0–20 cm depth than at the 20–40 cm depth. Similar results were found in flood irrigation studies^34^. The proportion of the root system in the 20–40 cm depth was greater in W1 than in either W2 or W3. This indicated that W1 could lead to better use of stored soil water. The amount of water at the 0–20 cm depth was not enough for plant growth when irrigation was less frequent. Root length increased when the plants were irrigated less frequently. This enabled plants to take up water from lower soil depths.

Roots grow long because roots can use deep water in soil. Our research suggested that the majority of wheat roots were in the 0–20 cm depth in this drip-irrigated system. However, wheat responded to drier soil conditions in W1 by increasing the root length at 20–40 cm depth. Our study showed that SRL had different performances in the two cultivars. This may be caused by the different changes in root morphology and structure. Research suggests that a higher SRL would be associated with increases in the number of growing root tips and thinner secondary branches^35^.

**Figure 9:**
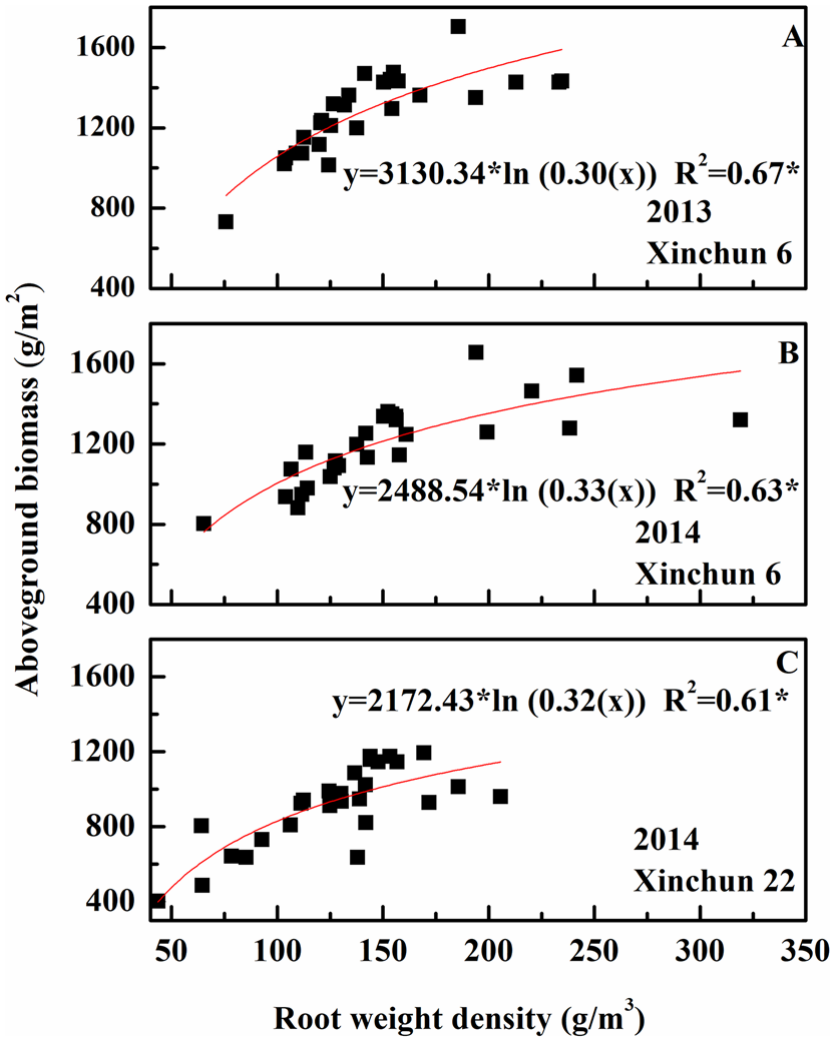
Relationship between root weight density and aboveground biomass in 2013 and 2014. (**A**) Relationship between root weight density and aboveground biomass of Xinchun 6 in 2013. (**B**) Relationship between root weight density and aboveground biomass of Xinchun 6 in 2014. (**C**) Relationship between root weight density and aboveground biomass of Xinchun 22 in 2014.

Drought-induced changes in the root-shoot ratio can indicate drought tolerance. Drought stress reduces wheat yield, root growth, and shoot dry weight and increases the root-shoot ratio^36,37^. It was similar with our study. Some researchers have reported a highly significant positive correlation between root dry weight and shoot dry weight. We observed no correlation between RWD and leaf dry matter, shoot dry matter, or spike dry matter (data not shown). However, there was a significant positive correlation between RWD and aboveground biomass (Fig. 9).

Photosynthesis is the basis of biomass accumulation and yield^38^. Water stress is an important factor affecting photosynthesis^39^. Previous studies indicated that moderate and severe water stress reduced Pn in the flag leaf of spring wheat^40^. Similarly, flag leaf Pn in this study decreased as irrigation frequency and irrigation amount decreased (Fig. 7). Some studies indicate that water stress increases abscisic acid concentrations in xylem, resulting in stomatal closure^41^. Other researchers report that Pn decreases due to nonstomatal factors. Specifically, progressive downregulation or inhibition of metabolic processes leads to decreased RuBP content, which inhibits photosynthetic CO_2_ assimilation^42^. Additional studies are required to determine the mechanism that caused the decreases in Pn as irrigation frequency and irrigation amount decreased.

Grain filling in wheat depends on two main sources: (1) current assimilates produced by leaf and stem photosynthesis and (2) mobilization of stored carbohydrates and N^43^. The dry weights at flowering decreased in the order stem > leaf > spike. There was no significant difference in plant organ dry weight between W2 and W2. Irrigation frequency also had no significant effect on organ dry weight (Fig. 6). Previous studies have shown that wheat grain yield is closely related to dry matter accumulation, transport, and distribution after flowering^44^.

Root growth and distribution patterns have a major influence on crop production. Many researchers have studied the influence of roots on yield, water and nutrient absorption. Other studies have examined the effect of, as well as environmental and regulatory effects on root cultivation techniques^28^. Previous studies have shown that the amount of roots in deep soil must be increased to obtain greater rice and wheat yields^45^. Under drought stress, wheat mainly uses deep soil moisture to meet water demands^46^. Therefore, deep roots have an important influence on wheat yield, shallow root systems and the role of small middle roots, with no correlation between the total root and yield. We also wanted to determine the relationship between root characteristics and yield. In our research, RWD at 0–20 cm depth and RLD at 20–40 cm depth were correlated with drip-irrigated wheat yield (Fig. 8). However, the R squared values were less than 0.5. This may be because root growth at only one growth stage (flowering) was measured in this study. Flowering time is an important trait in wheat breeding, as it affects adaptation and yield potential^47^. We should conduct more research on different stages of root growth and determine the relationship between root growth and yield of drip-irrigated wheat in the future.

Due to the influence of geographical location, topography and landform, the spatial distribution of annual precipitation in Xinjiang is extremely uneven. Some studies indicate that from 1961 to 2013, the annual precipitation in all regions of Xinjiang showed a significant increasing trend. The temporal variation trend of precipitation in Xinjiang is bounded by 1986^48^. Before 1986, there was less precipitation, which was a dry period, and after 1986, there was more precipitation, which was a wet period. The monthly precipitation in northern Xinjiang shows a bimodal distribution, with the main peak appearing from May to July^49^, which happens to be the peak of water demand for spring wheat. Therefore, the irrigation volume and frequency set by this research will need to be adjusted appropriately in different years. This will also be a problem that we need to solve in our next work.

## Conclusion

Our results indicate that the RLD was greater in D1 and D2 than in D3 in the shallow soil layer and in deep soil. The RWD and root-shoot ratio were greater in D2 than in D1 and D3. Drip-irrigated wheat responded to drier soil conditions by increasing the proportion of roots deeper in the soil, so wheat maintained the majority of the roots near the soil surface regardless of water deficit stress. We found that with the optimum irrigation amount, increasing drip irrigation frequency increased wheat root length and root weight and increased aboveground biomass accumulation, thereby improving yield and water use efficiency.
